# Subclinical auditory effects of recreational diving: a multidimensional audiological assessment beyond the audiogram

**DOI:** 10.1007/s00405-026-10288-8

**Published:** 2026-05-05

**Authors:** Ahsen Kartal Özcan, Betül Yazar, Şahika Özer, Fatma Dutak, Kübra Aytaç, Ayşe Aslı Yılmaz Şahin

**Affiliations:** 1 Department of Audiology, Hamidiye Faculty of Health Sciences, University of Health Sciences, Tıbbiye Cad. No:38, Istanbul, Türkiye; 2 Department of Otorhinolaryngology, University of Health Sciences, Ümraniye Training and Research Hospital, Istanbul, Türkiye; 3 Department of Otorhinolaryngology, University of Health Sciences, Kartal Dr. Lütfi Kırdar City Hospital, Istanbul, Turkey

**Keywords:** Recreational diving, Wideband tympanometry, Distortion product otoacoustic emissions, Eustachian tube function, Middle ear resonance frequency

## Abstract

**Purpose:**

This study aimed to evaluate peripheral auditory function and Eustachian tube function in individuals engaged in recreational diving compared with non-diving controls, via a multidimensional audiological assessment approach.

**Methods:**

A total of 49 participants aged 18–50 years were included, comprising 24 individuals actively engaged in scuba or free diving who reported no auditory or neurotological complaints prior to initiating diving, and 25 non-diving controls without any otologic or neurologic disorders. All participants underwent otorhinolaryngologic examination, conventional and wideband tympanometry, Eustachian tube function (ETF) testing, pure-tone audiometry, and distortion product otoacoustic emission (DPOAE) measurements. The subjective ETF was assessed via the Eustachian Tube Dysfunction Questionnaire–7 (ETDQ-7).

**Results:**

Pure-tone audiometry, speech audiometry, and conventional tympanometric measurements were not significantly different between the groups (all *p* > 0.05). In the diver group, the middle ear resonance frequency was significantly lower in at least one ear than in the control group (*p* < 0.05). Objective ETF testing revealed a higher frequency of Eustachian tube dysfunction in diver group (*p* < 0.05), and ETDQ-7 scores were also significantly higher in this group (*p* < 0.05). Additionally, DPOAE measurements revealed significant group differences at higher frequencies, with reduced amplitudes observed in divers (*p* < 0.05).

**Conclusion:**

Recreational diving may be associated with subclinical alterations in middle ear mechanics, Eustachian tube function, and cochlear outer hair cell activity that are not detected by standard audiometric evaluations. The inclusion of wideband tympanometry, objective ETF testing, and high-frequency DPOAE measurements may provide complementary information for audiological evaluation of individuals engaged in recreational diving.

## Introduction

In recent years, the increasing popularity of recreational and professional diving activities has led to growing interest in their effects on human physiology and the associated health risks, particularly in relation to the auditory and vestibular systems [1, 2]. The environmental conditions encountered underwater, most notably changes in ambient pressure, can trigger various pathophysiological processes that affect the ear and its associated structures [1, 3].

The ear, as a fundamental component of the auditory and balance systems, is particularly sensitive to rapid pressure gradients that occur during the descent and ascent phases of diving. When pressure equalization is insufficient, a range of otologic and vestibular conditions may develop, including middle ear barotrauma, inner ear barotrauma, inner ear decompression sickness, and eustachian tube dysfunction [4–7]. Approximately 80% of diving-related complications are otolaryngologic in origin, with the majority involving the external, middle, and inner ear structures [1].

The findings regarding the effects of diving on the auditory system remain inconsistent in the literature. Several studies, particularly those involving professional and military divers, have reported an increased risk of hearing loss owing to repeated pressure exposure and accompanying environmental factors [4, 7, 8]. In contrast, long-term follow-up studies have suggested that diving does not impose additional risks beyond age-related auditory changes [8]. Moreover, investigations conducted in naval divers have indicated the absence of clinically detectable hearing loss on the basis of pure-tone audiometry, while emphasizing that conventional audiometric assessments may be insufficient to identify subclinical cochlear alterations [9]. Similarly, studies focusing on recreational divers reported preserved hearing thresholds without evidence of overt hearing loss [10].

The eustachian tube plays a crucial role in maintaining middle ear pressure equilibrium during diving, and dysfunction of this system has been linked to impaired pressure equalization and an increased risk of middle ear barotrauma [11]. However, studies that simultaneously assess both eustachian tube function and peripheral auditory function in divers are limited. Most existing studies have examined these systems independently, potentially overlooking subtle interactions and subclinical changes that may not be evident through conventional audiometric evaluations alone.

Therefore, the present study aimed to concurrently investigate peripheral auditory and eustachian tube function in individuals engaged in diving via a multidimensional audiological assessment approach. Specifically, we examined whether divers differ from non-diving controls in terms of peripheral auditory and eustachian tube function. Additionally, the potential effects of diving-related variables, including diving experience, duration of diving activity, total number of dives, and maximum diving depth, on these auditory outcomes were explored.

## Methods

### Study design and ethical approval

This cross-sectional observational study aimed to compare peripheral auditory function and eustachian tube function between divers and non-divers. The study was conducted at the Audiology and Speech Disorders Center of University of Health Sciences, Ümraniye Training and Research Hospital. Ethical approval was obtained from the Ümraniye Training and Research Hospital Clinical Research Ethics Committee (Date: June 23, 2022; Approval No: B.10.1TKH.4.34.H.GP.0.01/207). Written informed consent was obtained from all participants prior to their participation, and detailed information regarding the assessment procedures was provided.

### Participants

A total of 49 participants were included in the study, comprising 24 individuals actively engaged in scuba or free diving (mean age: 35.0 ± 5.47 years) and 25 non-diving individuals (mean age: 33.72 ± 7.38 years).

The inclusion criteria for the diver group included voluntary participation, age between 18 and 50 years, and active engagement in diving activities. Individuals who reported a history of hearing loss or hearing-related complaints at the time of their first dive were excluded. No additional prescreening criteria were applied for the assessment of peripheral auditory or eustachian tube function; all participants in the diver group were evaluated on the basis of their current clinical status. The control group was composed of non-diving individuals. The inclusion criteria for the control group were as follows: (a) bilateral pure-tone air-conduction thresholds ≤ 15 dB HL across the frequency range of 125–8000 Hz, (b) absence of middle ear pathology bilaterally (type A tympanogram), (c) presence of acoustic reflex responses at all tested frequencies between 500 and 4000 Hz, (d) presence of otoacoustic emission responses, and (e) absence of any known otologic or neurologic disorders.

Exclusion criteria for both groups included a known history of chronic occupational noise exposure and prior clinical ear barotrauma. These factors were specifically screened during the medical history interview to minimize their confounding effects on the audiological outcomes.

### Procedure

All participants underwent a standardized assessment protocol that included conventional tympanometry and wideband tympanometry (WBT), eustachian tube function testing (ETFT), pure-tone audiometry, and distortion product otoacoustic emission (DPOAE) testing. Prior to the audiological assessment, all participants underwent an otolaryngologic examination. In addition to these objective measures, all participants completed the Eustachian Tube Dysfunction Questionnaire–7 (ETDQ-7).

For the diver group, a detailed case history was obtained, including diving-specific variables such as duration of diving activity, total number of dives, and type of diving performed.

#### Acoustic immittance measures

Middle ear function was assessed via conventional tympanometry (226 Hz probe tone), wideband tympanometry (125 Hz–8 kHz frequency range), and acoustic reflex measurements with an Interacoustics Titan tympanometer (Interacoustics, Middelfart, Denmark). Middle ear pressure, ear canal volume, static acoustic compliance, and middle ear resonance frequency were evaluated in all participants. Resonance frequency values were classified according to the manufacturer’s recommendations, with 800–1200 Hz considered to be within normal limits. Values below 800 Hz were classified as having a reduced resonance frequency, and values above 1200 Hz were classified as having elevated resonance frequencies. The acoustic reflex thresholds were recorded ipsilaterally and contralaterally at 0.5, 1, 2, and 4 kHz.

#### Assessment of eustachian tube function

Eustachian tube function was evaluated via both objective and subjective methods. Objective assessment was performed via the Eustachian Tube Function (ETF) test, whereas subjective symptoms were assessed via the ETDQ-7.

The ETF test was conducted via an Interacoustics Titan tympanometer (Interacoustics, Middelfart, Denmark). Three consecutive tympanograms were recorded: the baseline tympanogram at rest (T1), the second tympanogram following the Valsalva maneuver (T2), and the third tympanogram following the Toynbee maneuver (T3). Eustachian tube function was considered adequate if either a ≥ 10 daPa change in middle ear pressure was observed between the T1 and T2 tympanograms or if the difference between the maximum and minimum pressure values was ≥ 15 daPa. These values were based on the cut-off criteria reported in previous studies that utilized the nine-step Eustachian tube function test [12, 13].

The ETDQ-7 is a self-report questionnaire consisting of seven items assessing the severity of symptoms related to Eustachian tube dysfunction. Each item is rated on a 7-point Likert scale, with higher total scores indicating more severe symptoms. The Turkish validity and reliability of the ETDQ-7 were established by Özgür, Bilgen, and Özyurt [14].

#### Pure-tone audiometry

Hearing assessment was performed via a GN Otometrics Madsen Astera² clinical audiometer (GN Otometrics, Denmark) in a sound-treated booth that was compliant with international standards. Air-conduction thresholds were obtained at frequencies between 125 and 8000 Hz, and bone-conduction thresholds were assessed between 500 and 4000 Hz.

The pure-tone average (PTA) was calculated by hearing thresholds of 500, 1000, 2000, and 4000 Hz and was used for the statistical analyses. Audiometric findings were classified according to Clark’s classification of hearing loss.

#### Otoacoustic emissions

DPOAE measurements were obtained via the Otodynamics Echoport ILO292-II system (Otodynamics, Hatfield, United Kingdom). Bilateral recordings were collected at frequencies of 1, 1.4, 2, 2.8, 4, 6, and 8 kHz while the participants were seated comfortably and instructed to remain in a quiet environment. Prior to testing, the stimulus parameters were set to a f₂/f₁ ratio of 1.22, with primary tone levels of L₁ = 65 dB SPL and L₂ = 55 dB SPL. A DPOAE response was considered to be present if the signal-to-noise ratio was ≥ 6 dB at a minimum of three frequencies.

### Statistical analysis

An a priori power analysis was conducted using G*Power (version 3.1) to determine the minimum required sample size. Based on the mean resonance frequency values reported by Stieler et al. [16], the estimated effect size was Cohen’s d = 0.95. With a significance level of α = 0.05 and a statistical power of 0.80, the required minimum sample size was calculated as total 38 participants. The present study included 49 participants, which exceeded the minimum sample size required to detect group differences.

Statistical analyses were performed via IBM SPSS Statistics version 27.0 (IBM Corp., Armonk, NY, USA), and graphical representations were generated via JASP version 0.95.1 (University of Amsterdam, Amsterdam, The Netherlands). Descriptive statistics are reported as the means ± standard deviations for normally distributed variables, medians (interquartile ranges [IQRs]) for non-normally distributed variables, and frequencies and percentages (n, %) for categorical variables.

All analyses were conducted separately for the right and left ears. The normality of the data distribution was assessed via the Shapiro–Wilk test. As the data did not meet normality assumptions, group comparisons between divers and controls were performed via the Mann–Whitney U test. Categorical variables were analyzed via Pearson’s chi-square test, and Fisher–Freeman–Halton exact test was applied when the expected cell counts were insufficient. Associations between the variables were examined via Spearman’s rank-order correlation coefficients. The correlation strength was determined according to the classification proposed by Evans [15]. Statistical significance was set at *p* < 0.05 for all analyses.

## Results

Fourteen participants in the control group were men (56%) and 11 were women (44%). The diver group consisted of 24 participants, with 14 men (58.3%) and 10 women (41.7%). Specifically, the diver group included five free divers (all women) and 19 scuba divers (14 men and 5 women). No statistically significant difference was observed between the diver and control groups in terms of mean age (t(47) = − 0.89, *p* = 0.379).

The auditory and vestibular symptoms that occurred before, during, and after diving were evaluated on the basis of the medical history of the divers. None of the divers reported a history of hearing- or balance-related complaints prior to initiating diving activities. During diving, two divers (8.3%) reported subjective hearing loss, whereas five divers (20.8%) reported hearing-related complaints after diving. With respect to vestibular symptoms, fourteen divers (58.3%) reported balance problems while diving, and four divers (16.7%) reported such symptoms after diving.

Descriptive characteristics of the diver group showed a median diving experience of 6.0 years (IQR: 14.25; range: 1–30). Participants performed a median of 2 dives per week (IQR: 2; range: 1–6), with a median dive duration of 40 min (IQR: 35; range: 30–240). The median maximum depth reached was 30 m (IQR: 15; range: 5–50), and the median total number of dives was 275 (IQR: 627.5; range: 10–6000).

### Pure-tone and speech audiometry findings

**Fig. 1:**
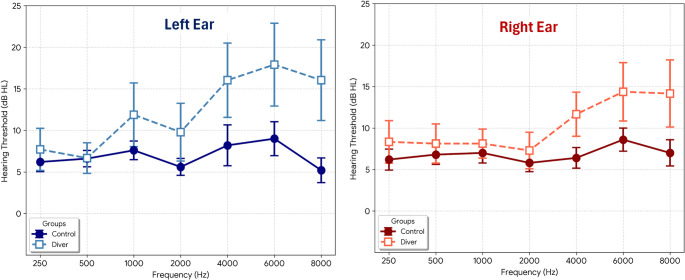
Comparison of pure-tone air conduction thresholds between diver and control groups across frequencies for right and left ears

No statistically significant differences were found between the diver and control groups in bilateral air conduction pure-tone thresholds across all frequencies (*p* > 0.05; Fig. 1). Similarly, no significant differences were observed between the groups in bilateral air- and bone-conduction PTA (*p* > 0.05). No significant between-group differences were observed in speech audiometry measures, including speech recognition thresholds and speech discrimination scores.

### Middle ear and acoustic reflex findings

No statistically significant differences were found between the groups for middle ear pressure, ear canal volume, or static acoustic compliance in either ear (*p* > 0.05; Table 1). However, when middle ear resonant frequencies were compared, the diver group demonstrated significantly lower resonant frequencies in the right ear than did the control group (z = − 2.380, *p* = 0.017). No significant difference was observed in the left ear (z = − 1.600, *p* = 0.110; Table 1).

**Table 1:** Comparison of tympanometric findings, middle ear resonant frequencies, and acoustic reflex thresholds between groups

	Tests	Groups	n	Min – Max	Median (IQR)	z	p
Tympanometry	**RE MEP (daPa)**	**Divers**	24	-119–132	-11.00 (36.75)	-1.271	0.204
		**Controls**	25	-68–37	-5.00 (15.00)		
	**LE MEP (daPa)**	**Divers**	24	-367–53	-11.00 (37.25)	-1.231	0.218
		**Controls**	25	-85–61	-8.00 (17.00)		
	**RE ECV (mL)**	**Divers**	24	0.67–2.18	1.34 (0.50)	-1.671	0.095
		**Controls**	25	0.10–2.07	1.01 (0.42)		
	**LE ECV (mL)**	**Divers**	24	0.55–2.50	1.24 (0.50)	-1.641	0.101
		**Controls**	25	0.64–2.33	1.02 (0.45)		
	**RE SC (mL)**	**Divers**	24	0.20–1.81	0.60 (0.35)	-0.575	0.565
		**Controls**	25	0.30–1.30	0.50 (0.40)		
	**LE SC (mL)**	**Divers**	24	0.20–1.90	0.57 (0.59)	-1.228	0.219
		**Controls**	25	0.30–1.27	0.52 (0.20)		
	**RE RF (Hz)**	**Divers**	24	460–1225	764.50 (235.75)	-2.380	0.017*
		**Controls**	25	802–1071	871.00 (58.00)		
	**LE RF (Hz)**	**Divers**	24	252–1549	790.00 (324.25)	-1.600	0.110
		**Controls**	25	813–1009	885.00 (64.00)		
Acoustic Reflex	**Right 500**	**Divers**	21	80–100	85.00 (12.50)	-0.749	0.454
		**Controls**	25	80–100	90.00 (7.50)		
	**Right 1000**	**Divers**	22	80–95	85.00 (10.00)	-0.388	0.698
		**Controls**	25	80–95	85.00 (5.00)		
	**Right 2000**	**Divers**	23	80–95	90.00 (10.00)	-0.627	0.531
		**Controls**	25	80–100	90.00 (10.00)		
	**Right 4000**	**Divers**	17	80–100	90.00 (15.00)	-0.891	0.373
		**Controls**	25	80–100	90.00 (10.00)		
	**Left 500**	**Divers**	21	80–95	85.00 (12.50)	-0.569	0.569
		**Controls**	25	80–100	90.00 (7.50)		
	**Left 1000**	**Divers**	22	80–95	85.00 (7.50)	-0.861	0.389
		**Controls**	25	80–100	85.00 (12.50)		
	**Left 2000**	**Divers**	22	80–95	85.00 (10.00)	-0.640	0.522
		**Controls**	25	80–100	85.00 (12.50)		
	**Left 4000**	**Divers**	17	80–100	85.00 (15.00)	-0.472	0.637
		**Controls**	25	80–100	90.00 (12.50)		

*RE,* Right ear; *LE*, Lefte ear; *MEP*, Middle ear pressure; *ECV,* Ear canal volume; *SC,* Static Compliance; *n*, number of participants; *Min,* Minimum; *Max,* Maximum, *Indicates statistical significance (*p* < 0.05)

Acoustic reflex responses were obtained from all the control participants. In the diver group, however, acoustic reflexes were absent bilaterally at 0.5, 1, and 2 kHz in three participants, and at 4 kHz in seven participants. Despite this, no statistically significant differences were observed between the groups in bilateral acoustic reflex thresholds across frequencies (*p* > 0.05).

When the resonant frequencies were analyzed categorically (normal, decreased, and increased), all control participants presented bilateral resonant frequencies within normal limits. In contrast, reduced or increased resonant frequencies were observed in 62.5% of the right ears and 54.2% of the left ears in the diver group (Table 2). These differences were statistically significant for both ears (right ear: *p* < 0.001; left ear: *p* < 0.001).

**Table 2:** Distribution of middle ear resonant frequency categories by groups

Ear	Group	*n*	Decreased *n* (%)	Normal *n* (%)	Increased *n* (%)	χ²	*p*
Right	**Divers**	24	14 (58.3%)	9 (37.5%)	1 (4.2%)	24.828	< 0.001*
	**Controls**	25	0 (0.0%)	25 (100.0%)	0 (0.0%)		
Left	**Divers**	24	12 (50.0%)	11 (45.8%)	1 (4.2%)	20.048	< 0.001*
	**Controls**	25	0 (0.0%)	25 (100.0%)	0 (0.0%)		

n: Number of participants, χ²: Chi-square value. *Indicates statistical significance (*p* < 0.05). Fisher-Freeman-Halton Exact Test was used

### Eustachian tube function findings

The ETF was evaluated via two pressure-change criteria (Table 3). According to both criteria, all control participants demonstrated functional eustachian tubes. In the diver group, the Eustachian tube dysfunction rates based on the 10 daPa criterion were 29.2% for the right ear and 41.7% for the left ear, which were significantly higher than those of the control group (*p* < 0.05). When using the 15 daPa criterion was used, bilateral eustachian tube dysfunction was observed in 20.8% of divers, with a significant difference between the groups (*p* = 0.022).

**Table 3:** Comparison of the Eustachian tube function test results based on the 10 daPa and 15 daPa criteria

Ear	Criterion	Group	*n*	Functional *n* (%)	Dysfunction *n* (%)	*p*
Right	**10 daPa**	**Divers**	24	17 (70.8%)	7 (29.2%)	0.004*
		**Controls**	25	25 (100.0%)	0 (0.0%)	
	**15 daPa**	**Divers**	24	19 (79.2%)	5 (20.8%)	0.022*
		**Controls**	25	25 (100.0%)	0 (0.0%)	
Left	**10 daPa**	**Divers**	24	14 (58.3%)	10 (41.7%)	< 0.001*
		**Controls**	25	25 (100.0%)	0 (0.0%)	
	**15 daPa**	**Divers**	24	19 (79.2%)	5 (20.8%)	0.022*
		**Controls**	25	25 (100.0%)	0 (0.0%)	

*Indicates statistical significance (*p* < 0.05). Fisher-Freeman-Halton Exact Test was used

Subjectively, ETDQ-7 scores were significantly higher in the diver group (median [IQR]: 11.50 [6.50]) than in the control group (7.00 [0.00]; *p* < 0.001).

### Otoacoustic emission findings

In the control group, DPOAEs were present bilaterally at all frequencies (SNR ≥ 6 dB) in all participants. In the diver group, DPOAEs were present at a minimum of three frequencies in 20 participants, whereas no measurable DPOAE responses were obtained in four participants.

**Fig. 2:**
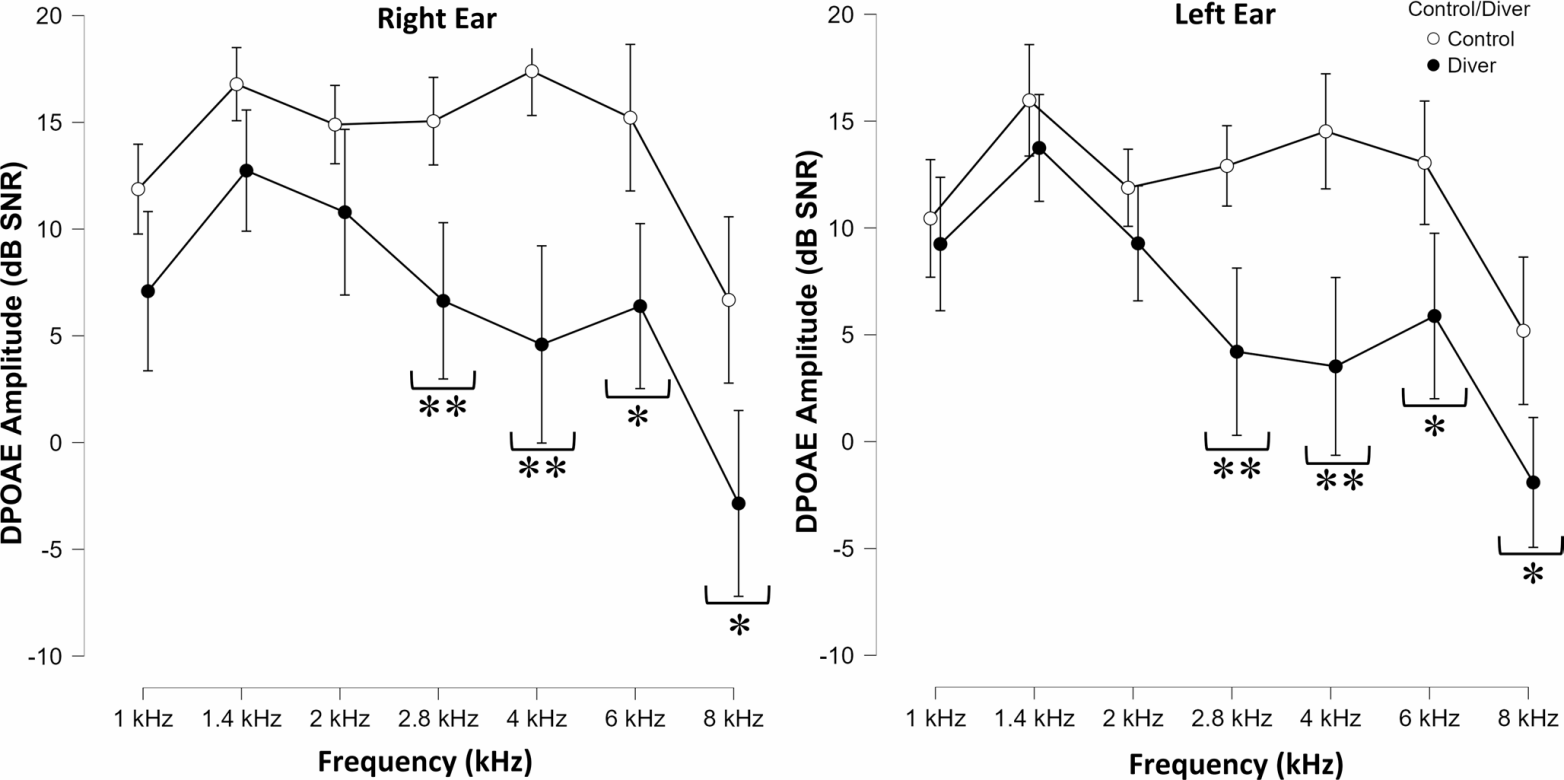
Comparison of DPOAE amplitudes between diver and control groups across frequencies for right and left ears. The error bars represent 95% Confidence Intervals (CIs). (* *p* < 0.05, ** *p* < 0.01)

When the overall DPOAE results were compared between the groups, no significant differences were observed bilaterally (*p* > 0.05). However, frequency-specific analysis revealed significantly lower DPOAE amplitudes in the diver group at 2.8, 4, 6, and 8 kHz in both ears (*p* < 0.05; Fig. 2).

### Correlation analysis in the diver group

No statistically significant correlations were found between diving-related variables (diving experience, weekly dive frequency, dive duration, dive depth, and total number of dives) and hearing thresholds, PTAs, tympanometry parameters, acoustic reflex thresholds, DPOAE SNR values, or ETDQ-7 scores (*p* > 0.05).

However, moderate negative correlations were observed between diving experience and resonant frequencies in both the right (*r* = − 0.413, *p* = 0.045) and left ears (*r* = − 0.429, *p* = 0.037). Additionally, moderate positive correlations were found between dive duration and bilateral acoustic reflex thresholds at 500, 1000, and 2000 Hz (*p* < 0.05).

## Discussion

In this study, peripheral auditory function and the ETF in divers were examined via a multidimensional assessment approach. These findings indicate that diving-related auditory changes may occur at a subclinical level prior to the development of clinically detectable hearing loss. A review of the literature revealed that auditory effects in divers have most commonly been evaluated via pure-tone audiometry and conventional tympanometry, whereas studies that simultaneously incorporate more sensitive parameters such as the middle ear resonance frequency and ETF remain limited. In this context, the present study contributes to the literature by providing a more comprehensive evaluation of the multiple components of the auditory system within the same sample, thereby allowing a more detailed characterization of diving-related subclinical changes.

No statistically significant differences were observed between the diver and control groups in terms of pure-tone hearing thresholds or PTAs. This finding is consistent with previous studies reporting preserved hearing thresholds in recreational and sport divers [5, 8, 10]. In particular, long-term follow-up studies suggest that diving does not pose an additional risk beyond age-related hearing changes, which is in line with the normal audiometric thresholds observed in the present study [8]. In contrast, studies involving professional and military divers have reported elevated hearing thresholds, particularly at high frequencies, along with increased hearing-related symptoms [4, 7]. These discrepancies may be attributed to differences in the diving type, exposure duration, diving depth, and concurrent noise exposure. Compared with recreational diving, the operational conditions and equipment used in military and professional diving may impose additional auditory loads [6].

Similarly, no significant between-group differences were found in middle ear pressure, ear canal volume, or static acoustic compliance as assessed by conventional tympanometry. This finding suggests that diving-related middle ear involvement may not always be detectable via standard tympanometric parameters. Indeed, previous studies have reported normal tympanometric findings in divers without overt middle ear barotrauma [3]. Likewise, the absence of significant differences in acoustic reflex measures indicates relative preservation of the middle ear–brainstem reflex pathway, supporting the notion that diving-related auditory effects may not yet manifest at the reflex level.

In contrast to conventional tympanometric findings, middle ear resonance frequency analysis revealed a more sensitive indication of diving-related effects. Resonance frequencies were significantly lower in the divers, particularly in the right ear, and the proportion of individuals with values outside the normal range was significantly higher than that in the controls. These findings suggest that repeated exposure to pressure changes during diving may influence the mass–stiffness balance of the middle ear system, leading to subclinical mechanical alterations despite the absence of overt middle ear pathology detectable by conventional methods.

Our findings are consistent with those reported by Stieler et al. [16], who reported reduced resonance frequencies in regular divers despite normal results on conventional audiological tests. Moreover, compared with the resonance frequency values reported for the adult Turkish population by Polat et al. [17] (933 ± 250.21 Hz in males and 992.45 ± 215.97 Hz in females), the resonance frequencies observed in the diver group in the present study were closer to or below the lower limit of these reference ranges. This pattern suggests a relative increase in mass-related effects within the middle ear. Taken together, these results support the use of resonance frequency measurements obtained via wideband tympanometry as a sensitive marker for detecting subclinical middle ear changes associated with diving.

In this study, ETF was assessed via both objective (ETF test) and subjective (ETDQ-7) methods. Findings indicative of Eustachian tube dysfunction were observed more frequently in the diver group than in the control group, suggesting that repeated exposure to pressure changes during diving may adversely affect the ETF. Given the critical role of the eustachian tube in middle ear pressure regulation, impaired function may increase the risk of pressure equalization difficulties and middle ear barotrauma [3]. In a recent study, Quarato et al. [11] reported comparable ETDQ-7 scores between navy divers and controls; however, they also noted an increased risk of Eustachian tube dysfunction in divers that was not associated with the duration or frequency of diving activity. When considering the resonance frequency changes observed in the present study, our findings suggest that diving-related effects may influence the middle ear–eustachian tube system as a whole. These results underscore the importance of incorporating eustachian tube function assessment into comprehensive auditory evaluations of divers rather than relying solely on pure-tone audiometry.

With respect to otoacoustic emissions, previous studies involving recreational, military, and professional divers have reported heterogeneous findings [9, 10]. This variability has been attributed to methodological factors, including the type of otoacoustic emission measured (TEOAE vs. DPOAE), timing of assessment, and exposure characteristics. In the present study, the use of high-frequency, sensitive DPOAE measures, together with simultaneous assessment of middle ear characteristics, enabled the detection of subtle cochlear changes associated with diving.

Although pure-tone hearing thresholds were preserved, a trend toward reduced DPOAE amplitudes at higher frequencies was observed in the diver group. These findings provide important insights into the pathophysiology of diving-related cochlear involvement. Previous studies have suggested that intravascular or intralabyrinthine gas bubbles formed during diving may disrupt the vascular circulation of the inner ear, leading to hypoxic injury or microhemorrhages due to compromised vascular integrity [1, 2]. Given the increased vulnerability of the basal turn of the cochlea to vascular compromise, the observed reduction in high-frequency DPOAE amplitudes may reflect early functional alterations in outer hair cell activity. This pattern likely represents subclinical cochlear involvement that is not yet detectable via pure-tone audiometry. Accordingly, reliance solely on audiometric thresholds may overlook early cochlear changes, whereas DPOAE measures may serve as valuable complementary tools in the preventive auditory monitoring of divers.

Finally, correlation analyses revealed no significant associations between most diving-related variables and auditory parameters, suggesting that diving-related auditory effects may not follow a simple linear exposure–response relationship. However, moderate correlations between diving experience and resonance frequency, as well as between diving duration and selected acoustic reflex thresholds, indicate that cumulative pressure exposure may influence middle ear mechanical properties and reflex responses over time. The modest strength of these associations further suggests that individual susceptibility and multifactorial influences are likely to play a role in diving-related auditory outcomes. However, the absence of statistically significant correlations should be interpreted with caution, as correlation analyses generally require larger samples. Therefore, the lack of significant relationships between diving exposure variables and auditory outcomes observed in the present study may partly reflect limited statistical power rather than the complete absence of an association.

This study has several limitations. First, its cross-sectional design precludes the evaluation of longitudinal changes and limits causal inferences. Second, the relatively small sample size may have reduced the statistical power, particularly for correlation analyses involving diving-specific variables. Additionally, diving-related data were obtained via self-reports, which may have introduced recall bias regarding diving depth, frequency, and duration. Furthermore, smoking status was not recorded during data collection, which should be considered a limitation given its potential impact on eustachian tube function. Finally, only auditory status at the time of assessment was evaluated, precluding the analysis of short- and long-term post-dive changes. Despite these limitations, a key strength of this study lies in its comprehensive and multidimensional assessment of peripheral auditory function, middle ear resonance characteristics, and eustachian tube function within the same sample. These findings provide a valuable foundation for future prospective, longitudinal studies.

## Conclusion

This study demonstrated that even when pure-tone audiometry and conventional tympanometry findings remain within normal limits, subclinical alterations in auditory and Eustachian tube function may be present in individuals who engage in diving activities. The reduced resonance frequencies identified through wideband tympanometry suggest that repeated pressure exposure may influence middle ear biomechanics by altering the mass–stiffness balance, whereas trends toward reduced high-frequency DPOAE amplitudes indicate early functional cochlear involvement. In addition, the greater prevalence of findings suggestive of Eustachian tube dysfunction among divers highlights the importance of middle ear pressure regulation in the assessment of diving-related auditory risk. Taken together, these results suggest that relying solely on pure-tone audiometry may overlook early auditory changes in divers. Multidimensional assessment approaches incorporating wideband tympanometry, Eustachian tube function testing, and high-frequency DPOAE measurements may therefore provide clinically relevant complementary information for the audiological evaluation and monitoring of individuals engaged in diving activities.

## Data Availability

The data that support the findings of this study are available from the corresponding author upon reasonable request.
